# Mathematical Modeling of Goat Meat Drying Kinetics with Thermal Oscillations

**DOI:** 10.3390/foods13233836

**Published:** 2024-11-28

**Authors:** Valeria Carrillo Luis, Diego Beristain Rios, Omar Augusto Hernández-Flores, Carolina Romero-Salazar, Sadoth Sandoval-Torres

**Affiliations:** 1 Facultad de Sistemas Biológicos e Innovación Tecnológica, Universidad Autónoma Benito Juárez de Oaxaca, Av. Universidad s/n, Col. Cinco Señores, Oaxaca de Juárez 68120, Oaxaca, Mexicoohernandez.ciencias@uabjo.mx (O.A.H.-F.); cromero.cat@uabjo.mx (C.R.-S.); 2 Instituto Politécnico Nacional, CIIDIR-Unidad Oaxaca, Hornos No. 1003, Col. Noche Buena, Santa Cruz Xoxocotlán 71230, Oaxaca, Mexico

**Keywords:** thin layer theory, diffusion, heuristic model, mass transfer, fitting

## Abstract

Goat meat has a high nutritional value, since it contains up to 29% protein, as well as iron, potassium and vitamin B12. To prolong the shelf life of this food, a drying process can be applied; however, there is scarce information on the kinetics and drying parameters for this food material. The objective of this work was to characterize the thermal drying process of goat meat, through the mathematical modeling of convective drying kinetics, and its validation with experimental data obtained in a drying tunnel. The experiments were carried out with samples of loin (*Longissumus dorsi*) of Boer goat meat from the Mixteca region of Oaxaca (Mexico). Meat samples were subjected to air convection drying at 40, 50, 60 and 70 °C (with temperature oscillation), with air velocities of 1 and 2 m/s. Drying kinetics, air and meat temperature profiles, relative humidity and air flow velocity were recorded. Four models were analyzed: two-term, Midilli’s model, Wang and Singh’s model and a heuristic model with temperature dependence. The proposed mathematical models represented drying kinetics with an accurate fitting of experimental data, with standard errors (RMSE) in the range of 0.004–0.029. The estimated diffusion coefficients are consistent with the transport properties in biomaterials. The heuristic model was based on the solution of the effective diffusion equation considering the linearization of the temperature-dependent diffusion coefficient, showing a standard error in the range of 0.007–0.028, satisfactorily representing the temperature oscillations that allows a moisture diffusion reorganization, mainly when drastic temperature changes occur.

## 1. Introduction

The characterization and modelling of the convective drying of goat meat is important for the system design, quality improvement and optimization of drying processes [1]; nevertheless, there is scarce published work on this topic. Hot-air drying is a widely used drying technique for biological materials; however, in food drying, it is desirable that the process is promoted at low product temperatures in order to obtain better quality products [2,3,4]. According to Mediani et al., the consumption of dried meat is attractive for its nutritional value and quality [5]. The authors analyzed different types of dried meat and drying techniques, such as vacuum drying, ultrasound-assisted vacuum drying and freeze-drying. They also present an extensive description and comparison of physicochemical, microbiological and biochemical characteristics. Álvarez et al. mention that there is currently a wide variety of dried meat obtained from poultry, beef, pork, camel, sheep and goat [2]. Meat drying is often combined with other techniques such as smoking or fermentation, the use of brine, herbs, spices or vinegar, and depending on the culture and geographical region, the final product is known by different names such as Pastirma, Charki, Jerky, Cecina, Kilishi, Biltong, Carne-do-sol or Kaddid. However, because the nutrients in meat are sensitive to heat, a proper drying process is required to achieve a balance between drying temperature and quality. Therefore, to control and optimize the drying process, it is important to analyze the drying kinetics. In the work of Ahmat et al., the drying of meat of Goudali Zebu (4–5-year-old) was studied [6]. The round part of the hindquarters of the animal, without post-mortem treatment, was used. The authors applied three drying temperatures (10, 50, 60 °C)with three air flow rates (1, 2 and 3 m/s) and observed that temperature has a great impact on drying kinetics. They concluded that the Drying Characteristic Curve method and the Midilli’s model were the best to predict the drying curves. Morales-Cruz et al. conducted a study on the solar drying of goat meat. In the dryer, the air temperature was recorded, observing a variation from 35 to 51 °C [7]. Samples with a thickness between 3 and 5 mm were dehydrated. The results showed that the drying rate was not constant because the solar dehydrator is highly dependent on ambient conditions. To produce “Kaddid”, beef samples were subjected to an osmotic dehydration process prior to hot-air convection drying [8]. Kaddid is a product produced by a traditional process, where beef or lamb meat is cut into long pieces and salted in brine or dry salted. Meat fillets were obtained, with an approximate size of 5 cm × 2 cm × 0.5 cm, three temperatures were applied, 30, 40 and 50 °C and two air velocities (1.5 and 2.5 m/s). The authors found that pre-treatment at higher brine concentration slows down the drying kinetics. The drying rate was affected by brine concentration, air temperature and, to a lesser extent, by air velocity. Aykın-Dinçer et al. applied a vacuum drying process on beef slices [9]. The authors highlight that meat facilitates the growth of microorganisms due to its moisture content and water activity, hence the importance of removing moisture content to sufficiently low levels. Fat and visible connective tissue were removed, and the meat was cut into slices of 100 × 40 × 2 mm, parallel to the direction of the fiber. The slices were dried at 60, 70 and 80 °C to reach a final moisture content of 5%. The authors found that the hot-air-dried beef samples were significantly darker than the vacuum-dried ones. The study demonstrated that a dehydrated beef snack can be produced by applying vacuum drying in a temperature range of 60–80 °C. Mewa et al. studied the effect of drying air temperature and sample thickness on microbial quality and stability in beef [10]. Excess fat was removed to avoid rancidity during drying. The meat was cut in the direction of its fibers into thin strips of 100 mm length, 30 mm width and thicknesses of 2.5, 5.0, 7.5 and 10 mm. Four air temperatures were applied: 30, 40, 50 and 60 °C and airflow generated by a fan which was set at a constant voltage of 24 V. The experimental results showed that the drying temperature increased the value of the hue/H* colour parameter and decreased the value of the parameter L*, which may be associated with changes in the protein structure of the meat. The highest rehydration rate was observed in samples dried at 60 °C. The firmness of the meat showed non-monotonic behavior with respect to the drying temperature, with the meat with the lowest thickness being the one that showed the greatest softness. Kamiloğlu et al. conducted a comparative study between convection and microwave drying techniques for beef [11]. The meat samples were prepared in dimensions of 5 × 5 × 0.5 cm. For convective drying, three air temperatures (60, 70 and 80 °C) were applied; for microwave drying, three different powers (487, 686 and 889 W) were applied. Analyzing the drying kinetics, it was found that the drying time with microwaves was shorter. The effective diffusion coefficients and the activation energy were calculated for each kinetic. In the case of microwave drying, the effective diffusion coefficient was two orders of magnitude higher than that obtained for convective drying. Drãghici studied the effect of salting and acidification on the moisture content of dehydrated goat meat in a thermobalance at *T* = 103 °C [12]. The author used samples of 3 mm, which were mixed with distilled water (control sample), with brine and with lactic acid. The author evaluated different mathematical models for drying and found that the ‘Wang and Singh’ model best described the exponential variation in the moisture rate for all samples. Álvarez et al. studied the effect of beef sample geometry on drying dynamics [13]. They correlated moisture content, water activity, amount and type of moisture (bound, trapped and free) in different sections of the meat. The authors compared seven thin-layer models and used an exponential decay model with a constant phase. They determined the Midilli’s model was the most appropriate model to describe the drying during the ripening process. By lowering the moisture content, a reduction in microbial growth is accomplished. The reduced surface moisture plays a crucial role in preventing not only the growth of surviving bacteria but also the growth of any bacteria that may contaminate the surface of the product. The decrease in water activity inhibits the growth of contaminating microorganisms [14]. When controlling and optimizing a drying process, it is important to understand the drying kinetics, which describe the drying rate at specific conditions. According to Álvarez et al., a particular area of research in this field is mathematical modelling. Fitting a mathematical model capable of reproducing the drying kinetics is a powerful tool to understand and predict how drying conditions can impact moisture loss [2]. On the one hand, an air flow of 1 m/s would be a condition in which external resistances would be observed, so it is likely that the period of constant drying rate would be observed; on the other hand, an airflow of 2 m/s (or beyond) is typically used for meat drying, and in this case, internal resistances will be present. The four temperature levels were selected from the literature review, as they are temperatures that would prevent protein degradation [14].

In the present work, for the first time, the drying kinetics of Boer goat meat are presented. The drying parameters applying thermal oscillations were recorded. Four temperatures 40, 50, 60 and 70 °C and two air velocities 1 and 2 m/s were applied. The aim of the oscillations was to avoid excessive hardening of the meat. The aims of this study are as follows: (i) Obtaining hot-air-drying kinetics over a range of feasible conditions for goat meat, (ii) Measuring the temperature evolution of samples with air temperature oscillation, (iii) Identifying the magnitude of the drying rate and (iv) Implementing and solving the mathematical thin-layer models and a heuristic model for predicting the drying curves of goat meat.

## 2. Materials and Methods

### 2.1. Meat Samples and Preparation

Meat was obtained from the loin of a 3-year-old Boer goat (*Longissumus dorsi*) from the municipality of Coixtlahuaca in the Mixteca region (Oaxaca, México). The loin was frozen to facilitate slicing. Slices of meat were obtained, and the direction of cutting was parallel to the direction of the fibers. The slices were placed in plastic vacuum bags and stored in a freezer at a temperature of −17 °C. The thickness of the slices was 3–5 mm, while the length and width were 12 cm long and 4 cm, respectively.

### 2.2. Drying

**Figure 1:**
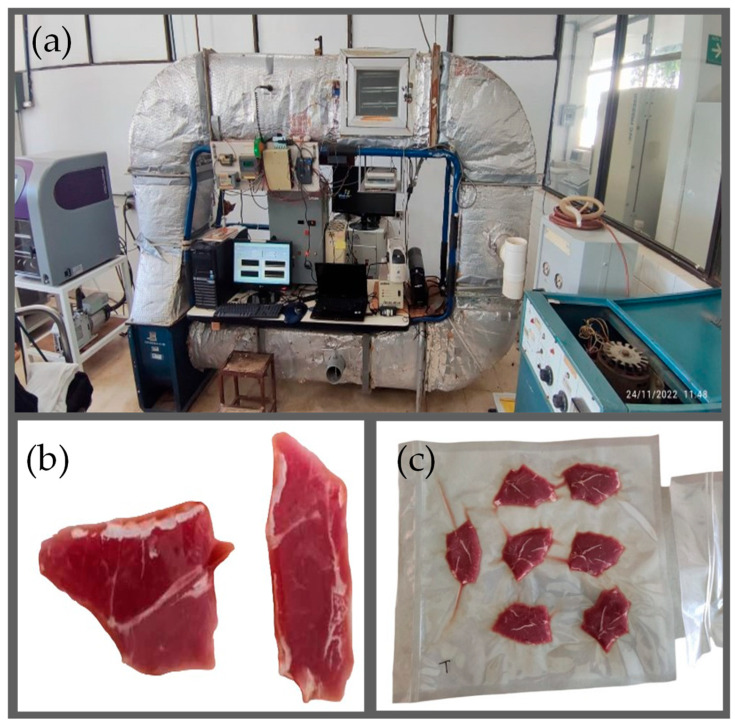
Drying facility and sample preparation. (**a**) Tunnel dryer. (**b**) Standard cut. (**c**) Vacuum packaging.

A cross-flow tunnel-type tray dryer (The Instituto Politécnico Nacional, Mexico City, Mexico) with patent number 304,462 was used. The dryer allowed temperature control by means of the myRIO-1900 card (National Instruments, Austin, TX, USA). Four central drying temperatures *T_c_* = 40, 50, 60, 70 °C and two air flow rates *v* = 1 m/s and *v* = 2 m/s were applied. Temperature oscillations were applied to allow the conditioning and reorganization of moisture diffusion. Before drying, the samples were placed in a refrigerator at a temperature of −4 °C. The meat samples were removed from their bags to be individually weighed and then placed on plastic nets in different trays. Three samples were placed on the first tray and four samples on the second tray. An Extech AN340 Anemometer (Nashua, NH, USA) was used to record the air velocity. Relative humidity measurement and *T*_Air_ temperature were recorded by a Vaisala HM70 (Helsinki, Finland) portable humidity and temperature meter. The surface and interior temperature of the meat samples were recorded using a Vaisala Veriteq 1700 data logger (Helsinki, Finland). Figure 1 shows the meat cuts and the drying tunnel.

### 2.3. Drying Kinetics

The moisture content *M* on a dry basis was obtained with the following expression:

(1) $$M\left(t\right)=\frac{m_{m}\left(t\right)}{m_{s}}=\frac{m\left(t\right)-m_{s}}{m_{s}},$$

where *m* is the total mass (wet matter), *m_m_* is the moisture mass and *m_s_* is the dry mass. The oven method (OAOC standard) was used to determine the initial moisture content. A Denver TP-214 analytical balance (accuracy 0.001 g) was used. The drying rate was calculated using regularized numerical differentiation [15].

### 2.4. Diffusion Coefficient

Usually, the diffusion coefficient is determined by a linear fit of the experimental MR moisture rate [16]; the slope of the fit line is related to the effective diffusion coefficient through Equation (2):

(2) $$\ln\left(\mathrm{MR}\right)=\ln\left(\frac{8}{\pi^{2}}\right)-D{\left(\frac{\pi }{L}\right)}^{2}t,$$

which is obtained by linearizing the first term of the general solution of the diffusion equation for an infinite plate of thickness *L*. The effective diffusion coefficient was estimated using the M1 (two-term) model; assuming the ratio of the fitting parameters satisfies the condition *k*_1_/*k*_2_ ≈ *a*_0_/*a*_1_ = 1/9, it is found that *k*_1_ = *D*(*π*/*L*)^2^. In the case of the temperature-dependent model, the effective diffusion coefficient was determined directly from the fitting parameters.

### 2.5. Activation Energy

The effective diffusion coefficient can be expressed as an Arrhenius-type equation [11,16,17] in which the exponential decay is affected by the activation energy *E_A_*:

(3) $$D\left(\theta \right)=D_{A}\exp\left\{-\frac{E_{A}}{k_{B}\theta }\right\}$$

where *θ* is the temperature in Kelvin, *k_B_* is the Boltzmann constant, *D_A_* is the effective diffusion for *θ* ≫ *θ_A_* = *E_A_/k_B_*. Note that *θ = T_S_* + 273 here *T_S_* is the temperature of the sample in degree Celsius. The activation energy and *D_A_* were obtained by linearizing Equation (4):

(4) $$\ln\left(\frac{D\left(\theta \right)}{D_{0}}\right)=\ln\left(\frac{D_{A}}{D_{0}}\right)-\frac{E_{A}}{k_{B}\theta }$$

For convenience, *D*_0_ is a constant of the same order of magnitude as the diffusion. The slope and the ordinate to the origin of the best fit line determine the values of *E_A_* and *D_A_*. For the case of the temperature-dependent model, the above parameters were obtained directly from the fitting process.

### 2.6. Mathematical Models

The mathematical models studied in this work are described in Table 1. The first three (M1, M2, M3) models are derived from approximations of the diffusion equation, Newton’s cooling law and polynomial regression, respectively. From the solution of the diffusion equation in the case of an infinite sample of thickness L [18], the model Heuristic + *R*_1_ (M4) is proposed, while the model Heuristic + *R*_1_ + *θ* (M5) is an extension of model M4 incorporating a temperature dependence. The models M1–M4 are isothermal approximations, thus a model that includes the effect of temperature is desirable. Juma Haydary and Noori, introduce a model sensitive to changes in the drying variables, i.e., to air temperature, air velocity, relative humidity and sample thickness, showing a good agreement with their experimental measurements of hot-air convective drying [19]. Their semi-empirical approach consists of modifying the solution of Newton’s cooling equation by incorporating dependent functions of the drying variables in the exponential decay. They assume that the sample is in thermal equilibrium with the air. Model M5 incorporates the average sample temperature *T_S_*, meaning that temperature controls the effective diffusion of moisture.

For comparison with experimental data, the reduced moisture content [29] is defined by Equation (5):

(5) $$\mathrm{MR}=\frac{M\left(t\right)-M_{f}}{M_{0}-M_{f}}$$

where *M*(*t*) is the moisture content at time *t*, *M*_0_ and *M_f_* are the initial and final moisture content, respectively. For long periods of drying, the moisture *M_f_* becomes the equilibrium moisture content *M_e_*. The fitting parameters of the models were found using the regression technique for non-linear models [30,31] using the Curve Fitting Toolbox in MATLAB.

In this paper, we introduce a heuristic model. A temperature-dependent moisture ratio MR was proposed. This model is based on the solution of the one-dimensional diffusion equation for infinite plate of thickness *L* and is described by Equation (6):

(6) $$\mathrm{MR}=\frac{8}{\pi^{2}}\left\{\exp\left\{-R_{3}f\left(\theta \right)t\right\}+\frac{1}{9}\exp\left\{-9R_{3}f\left(\theta \right)t\right\}+R_{2}-R_{1}t\right\}$$

where *R*_2_ = 0.1226, *R*_1_, *R*_3_ and *f*(*θ*) are defined by Equations (15), (19) and (20).

## 3. Results and Discussion

### 3.1. Convective Drying Kinetics Experiments

#### 3.1.1. Experiments at *v* = 1 m/s Airflow

**Figure 2:**
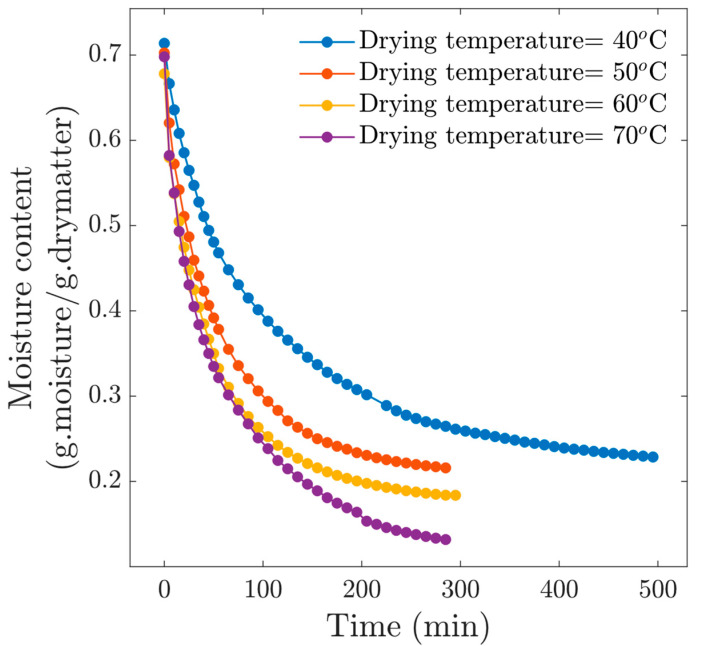
Drying kinetics for experiments at *v* = 1 m/s.

Figure 2 shows the drying kinetics at four drying temperatures, observing a standard behavior in this kind of process, i.e., the higher the temperature, the shorter the drying time. Likewise, the effect on the final moisture content is significant. At the drying time *t* = 285 min, we observed that the moisture content for the experiment at *T_c_* = 40 °C was *M* = 0.2646, *M* = 0.2158 at *T_c_* = 50 °C, *M* = 0.1838 at *T_c_* = 60 °C and *M* = 0.1318 at *T_c_* = 70 °C. Differences in final moisture content have implications for food preservation. About the drying condition *T_c_* = 40 °C, the drying time was excessively long, which exposed the material to deterioration, mainly in terms of hardening and decoloration. Moreover, drying at *T_c_* = 40 °C reached an equilibrium moisture content above 0.2 (d.b), which is very high for this type of product. From these results, we can observe that drying at 40 °C is not recommended.

**Figure 3:**
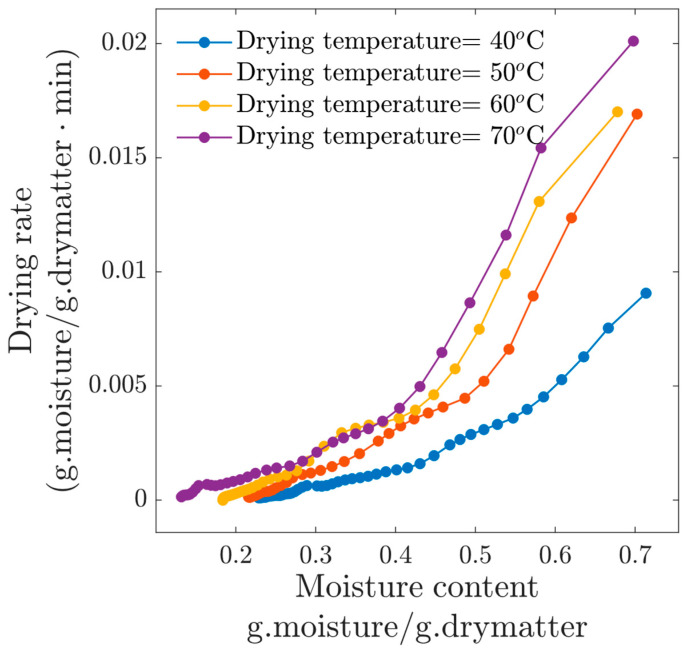
Drying rate for experiments at *v*=1 m/s.

The drying rate is related to the evaporation intensity at the surface of the product; therefore, in Figure 3, this information is presented for the four drying temperatures. The higher the drying temperature, the faster the drying rate, with a linear decay with respect to the moisture content. At the beginning of the experiments, the drying rate for the condition *T_c_* = 70 °C was close to 0.015 (d.b), which is approximately twice the initial drying rate for experiment at *T_c_* = 40 °C. Contrary to what some authors claim, the constant drying rate phase was not observed in our experiments at 1 m/s. In the work of Dinçer et al. (2020) [9], the authors mention that the drying of beef slices was largely controlled by the water inside the beef slices, and then drying occurs primarily during the falling rate and consequently is controlled by the internal diffusion phenomenon. This argument can be applied to our results, since according to Figure 3, the process was always in the falling drying rate period.

**Figure 4:**
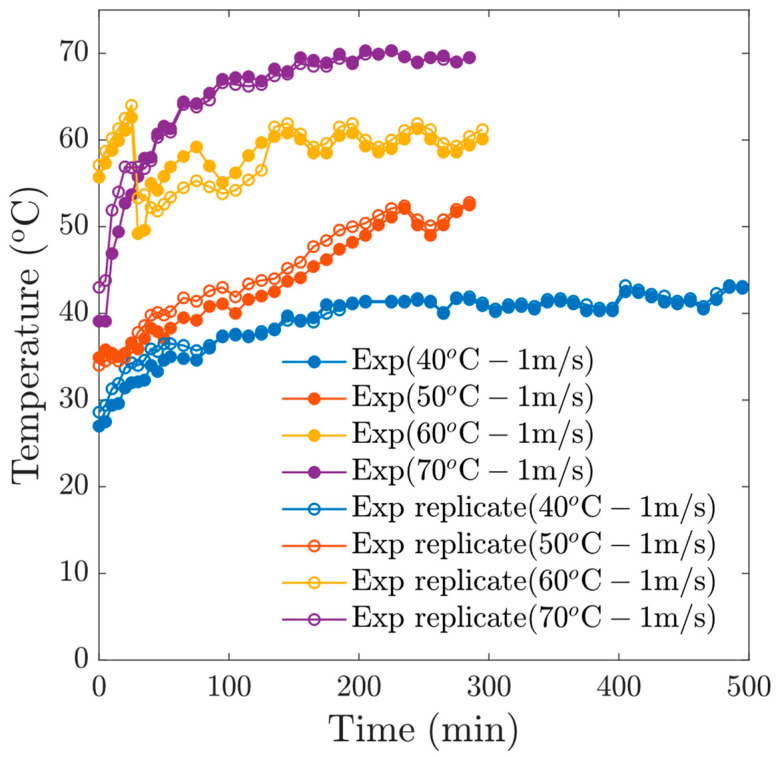
Thermal oscillations for experiments at *v* = 1 m/s.

In our experiments, thermal oscillations were applied, which allows a re-homogenization of the moisture at the surface of the samples. These heating patterns provide a relaxation of the tissue, which allows a better diffusion of moisture. Figure 4 shows the temperature profiles of the samples, which respond to the oscillatory pattern of the drying temperature.

#### 3.1.2. Experiments at *v* = 2 m/s Airflow

**Figure 5:**
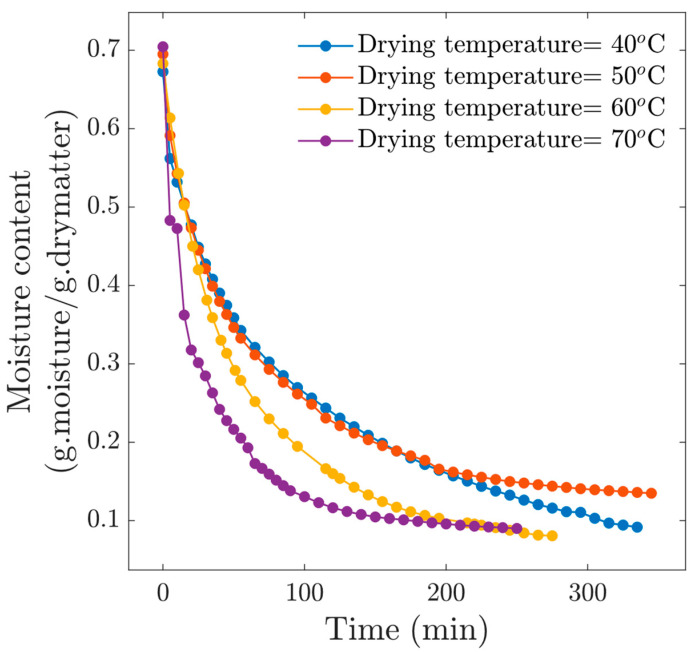
Drying kinetics for experiments at *v* = 2 m/s.

**Figure 6:**
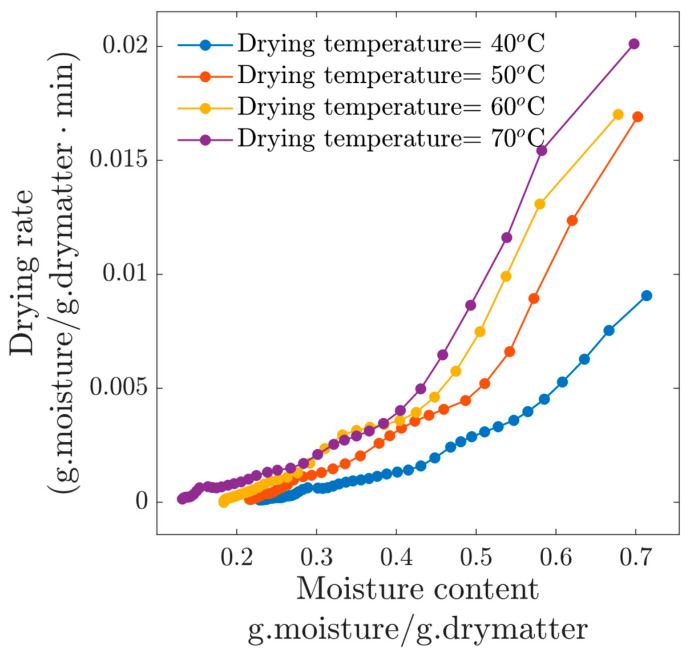
Drying rate for experiments at *v* = 2 m/s.

**Figure 7:**
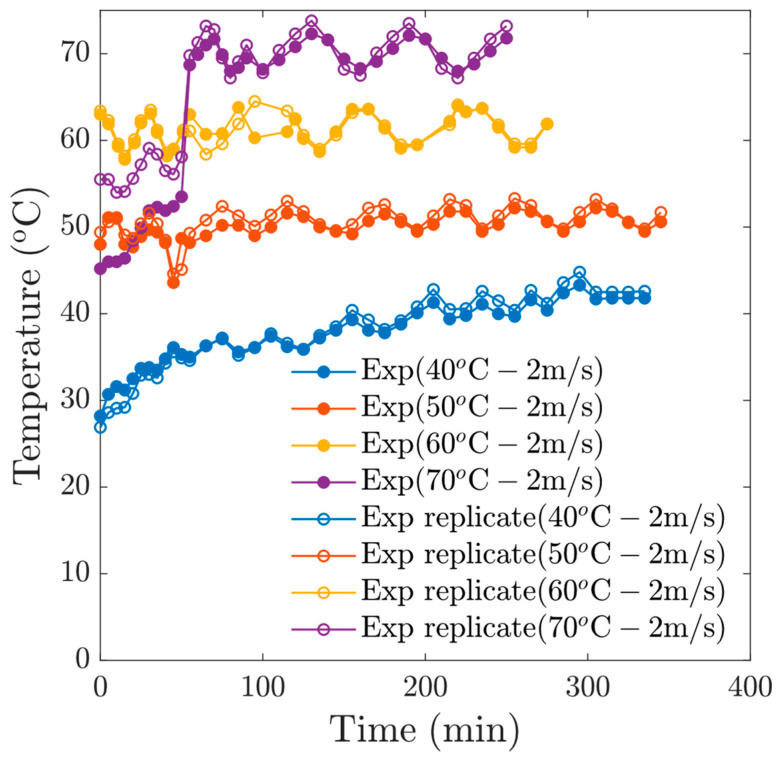
Thermal oscillations for experiments at *v* = 2 m/s.

Figure 5, Figure 6 and Figure 7 show the drying kinetics, the drying rate curves and the evolution of the temperature of the samples for the experiments at *v* = 2 m/s. The increase in the air flow speed influenced the drying kinetics, contrary to the drying of beef, where the drying rate was little affected by air velocity [8]. Nevertheless, goat meat and beef have very different biochemical properties. For these drying conditions, the drying kinetics reached a final moisture content below 0.2 (d.b). For experiments at *T_c_* = 60 °C and *T_c_* = 70 °C, the final moisture content was approximately 0.1 (d.b), which is much lower than that obtained for experiments at a *v* = 1 m/s. The temperatures of the samples are presented in Figure 6. The effect of the oscillation of the air temperature in the tunnel was important. A relaxation effect on the tissues improved the moisture diffusion in the samples. A significant effect of temperature was observed during the first 200 min. In the final phase of drying, an intersection of the curves was observed, which could be attributed to the effect of shrinkage, which modifies the moisture diffusion process. The shrinkage of meat is evident in this type of process, mainly when more severe drying conditions are applied, i.e., when the convection of the drying air increases. From this information, we can suggest that the temperature level should consider the shrinkage rate, and a subsequent analysis of the rheology of the material.

A theoretical drying rate curve for a porous product with high moisture content would show a short period of conditioning and an increasing drying rate, a period of constant drying rate and a period of decreasing drying rate. In our case, it is important to note that the drying process under our conditions exclusively reveals the falling drying rate. According to [2,6], internal resistance to mass transfer appears to predominate over external resistance to mass transfer when larger air-drying speeds are employed (beyond 2 m/s approximately). This would explain the absence of the constant drying rate period in Figure 6.

### 3.2. Implementation of Mathematical Models 

Three mathematical drying models were implemented (Table 2). The reduced moisture content at *t* = 0 was MR = 1, while as *M*(*t*) approaches *M_f_* the reduced moisture approaches zero. Model M1 represents models based on the diffusion equation, while model M2 represents a model derived from Newton’s cooling law, both from the framework of the thin-layer drying approach. The model M3 is a classical polynomial model, and linear regression was used to find its coefficients. Non-linear regression is an iterative procedure and therefore requires an initial estimation of the parameters. For the models M1 and M2, the asymptotic limit *M* → *M_f_* (MR → 0) was applied. For model M1, the parameters *b* ≈ 0.5, *k*_2_ ≈ *t_f_*^−1^, *k*_1_ ≈ 1.1*k*_2_ were calculated. With model M2, we obtained *b* ≈ MR(*t_f_*)*t_f_* ^−1^, *k* ≈ *t_f_*^−*n*^, *n* ≈ 1.

An important assumption for the thin-layer drying approach is that the initial local moisture content (or local concentration or scalar mass field) is uniformly distributed in the samples, i.e., the local moisture *M*(*x*,*t*=0) = constant [13]. However, in this type of heterogeneous multiphase materials, the reduction of dimensionality implies the impossibility of an approximation based on the spatial homogenization process, and with it, the dismissal of the homogeneity hypothesis.

**Figure 8:**
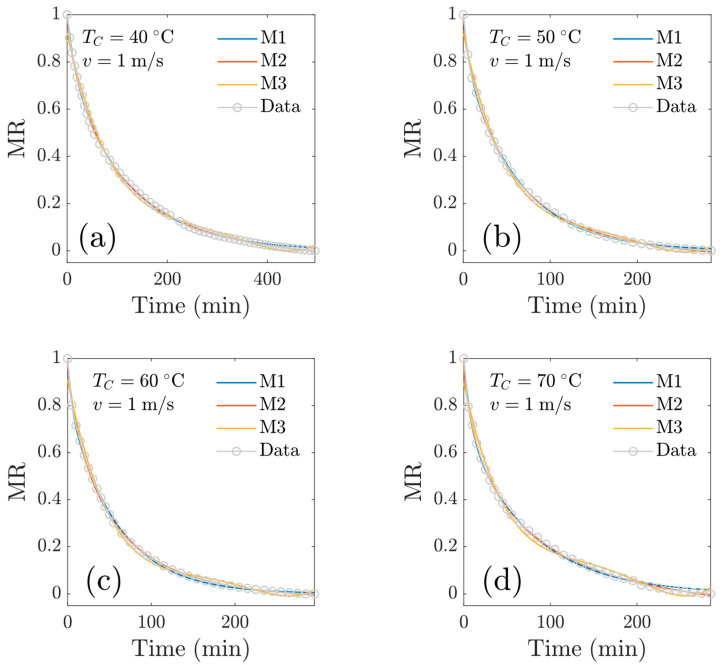
Reduced moisture content as a function of time at *v* = 1 m/s. Comparison of experimental data with three mathematical drying models: at a central temperature of (**a**) *T_c_* = 40 °C; (**b**) *T_c_* = 50 °C; (**c**) *T_c_* = 60 °C; (**d**) *T_c_* = 70 °C.

**Figure 9:**
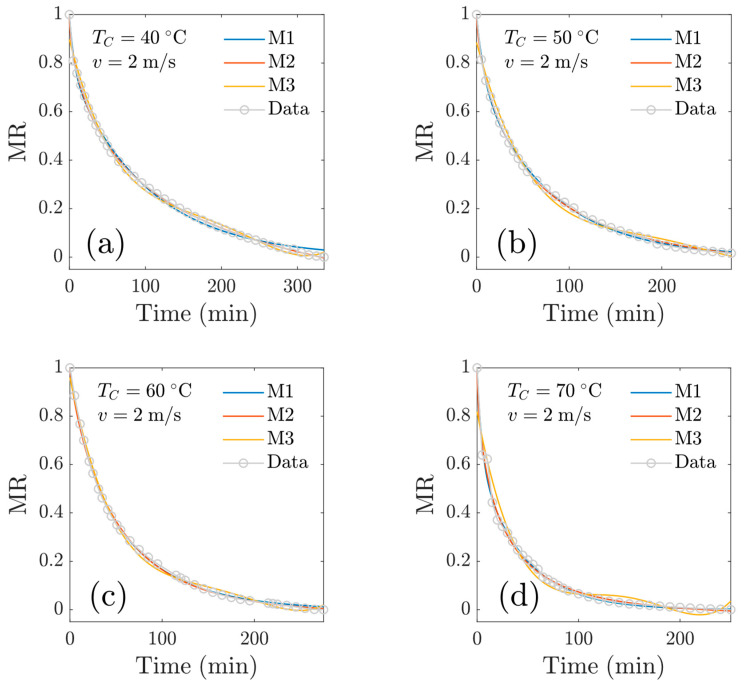
Reduced moisture content as a function of time at *v* = 2 m/s. Comparison of experimental data with three mathematical drying models: at a central temperature of (**a**) *T_c_* = 40 °C; (**b**) *T_c_* = 50 °C; (**c**) *T_c_* = 60 °C; (**d**) *T_c_* = 70 °C.

Figure 8 shows the modeled drying kinetics for experiments at *v* = 1 m/s. In general, a good fit was observed (to the essence of the statistical goodness-of-fit criteria). For the model M3, a polynomial of degree four was used. Models M1 and M2 describe the experimental data satisfactorily; furthermore, model M2 was better in terms of the goodness-of-fit criteria. Figure 9 shows the numerical results for experiments at *v* = 2 m/s, and again, model M2 was superior to model M1 in terms of goodness-of-fit criteria. The Midilli’s model (M2) showed good fitting of the experimental data, even in the presence of variations in temperature, findings that are consistent with the work of Ahmat et al., 2015 [6], who mention that the characteristic curve model and the Midili model were the best predictors of drying kinetics for beef samples. The M1 (two-term) model tends to the (physically correct) asymptotic limit MR=0 as time elapses. In the work of Drãghici (2021) [12], the author found the ‘Wang and Singh’ model best described the exponential variations in the moisture rate for drying goat meat; nevertheless, drying was conducted in a thermobalance at 103 °C (more drastic drying condition).

### 3.3. Effective Diffusion Coefficients from the Analytical Solution

The analytical solution of the diffusion equation for an infinite plate of thickness *L* is equivalent to the model M1, considering the first two terms of the analytical solution, in addition to the fact that *k*_1_/*k*_2_ ≈ *a*_0_/*a*_1_ = 1/9 y *b* = 1 − 8/*π*^2^ ≈ 0.19. This allows us to find the diffusion coefficient as $D=k_{1}{\left(L/\pi \right)}^{2}$. Table 3 summarises our results for the ratio *k*_1_/*k*_2_ and for the effective diffusion coefficients.

The activation energy *E_A_* is the minimum energy to remove water from biological material and its value is independent of temperature. According to our data and using Equation (3), we obtained *E_A_* ≈ 4–10 kJ/mol and *D_A_* ≈ 35 × 10^−8^ m^2^ s^−1^.

### 3.4. Heuristic Diffusion Model

This model considers temperature oscillations, and the diffusion coefficient depends on temperature. First, the solution of the one-dimensional diffusion equation for an infinite plate of thickness *L* is restructured and then the following conjecture is established: the MR obtained from the solution of the diffusion equation for the infinite plate maintains its functional form even when the diffusion varies with temperature.

#### 3.4.1. Isothermal Model of MR

The effective diffusion coefficient *D* was considered. The moisture content is described by the scalar mass field *MF*(*x*,*t*), where the spatial average is defined by *m_m_*(*t*) = 〈*MF*(*x*,*t*)〉. The 1D transient diffusion equation is as follows:

(7) $$\frac{\partial MF}{\partial t}=D\frac{\partial^{2}MF}{\partial x^{2}},$$

where initial and boundary conditions are *MF*(*x*,0) = *MF*_0_ (uniformly distributed) and *M*(0,*t*) = *MF*(*L*,*t*) = 0. Note that *m_m_*(0) = 〈*MF*_0_〉. The Equation (7) can be written as

(8) $$\frac{\partial MF^{\prime }}{\partial t}=D\frac{\partial^{2}MF^{\prime }}{\partial x^{2}},$$

where

(9) $$MF^{\prime }=\frac{\frac{MF}{MF_{0}}-\frac{M_{f}}{M_{0}}}{1-\frac{M_{f}}{M_{0}}}.$$

Here, *M_f_* and *M*_0_ are arbitrary constants chosen for convenience from the final moisture content and initial moisture content. The initial and boundary conditions are *MF*′(*x*,0) = 1 and *MF*′(0,*t*) = *MF*′(*L*,*t*) = 0. The analytical solution of Equation (8) is given by Equation (10) [18],

(10) $$MF^{\prime }\left(x,t\right)=\frac{4}{\pi }\overset{\infty }{\underset{n=0}{\sum }}\frac{1}{2n+1}\sin\left\{\left(2n+1\right)\frac{\pi x}{L}\right\}\exp\left\{-D{\left(2n+1\right)}^{2}\frac{\pi^{2}t}{L^{2}}\right\}.$$

The spatial average is

(11) $$\langle MF^{\prime }\rangle =\langle \frac{\frac{MF}{MF_{0}}-\frac{M_{f}}{M_{0}}}{1-\frac{M_{f}}{M_{0}}}\rangle =\frac{\frac{m_{m}\left(t\right)}{m_{m}\left(0\right)}-\frac{M_{f}}{M_{0}}}{1-\frac{M_{f}}{M_{0}}}=\frac{\frac{M}{M_{0}}-\frac{M_{f}}{M_{0}}}{1-\frac{M_{f}}{M_{0}}}=\frac{M-M_{f}}{M_{0}-M_{f}}=\mathrm{MR},$$

where *M* is the dry basis moisture content, *m_d_* the dry mass and *M_i_* and *M_f_* the initial and final dry basis moisture content, respectively. The spatial average of Equation (10) can be expressed as follows:

(12) $$\mathrm{MR}=\frac{8}{\pi^{2}}\overset{\infty }{\underset{n=0}{\sum }}\frac{1}{{\left(2n+1\right)}^{2}}\exp\left\{-a_{n}t\right\},$$

with *a_n_* = *D*(2*n* + 1)^2^ *π*^2^ *L*^−2^. The decay time of each exponential term is *τ_n_* = 1*⁄a_n_*; according to typical data of drying kinetics, diffusion has values around 10^−11^–10^−7^ m^2^ *t*^−1^ and *L =* 10^−3^ m, therefore, in the most precarious situation of diffusion, the first four decay times take the values *τ*_0_ = 166 min, *τ*_1_ = 18 min, *τ*_2_ = 6 min and *τ*_3_ = 3 min. MR is exact in three significant figures with the first five terms, while it is exact in one significant figure with the first two terms, then Equation (12) is written as follows

(13) $$\mathrm{MR}=\frac{8}{\pi^{2}}\left\{\exp\left\{-a_{0}t\right\}+\frac{1}{9}\exp\left\{-a_{1}t\right\}+R\left(t\right)\right\}$$

where *R* accumulates all the terms for *n* > 1. The term *R* can be written as

(14) $$R\left(t\right)=\overset{\infty }{\underset{n=2}{\sum }}\frac{1}{{\left(2n+1\right)}^{2}}\exp\left\{-a_{n}t\right\}\approx \overset{\infty }{\underset{n=2}{\sum }}\frac{1}{{\left(2n+1\right)}^{2}}\left\{e^{-a_{n}\tau }\left(1+a_{n}\tau \right)-ta_{n}e^{-a_{n}\tau }\right\}$$

Here, a Taylor series expansion was performed for the exponential. By defining

(15) $$R_{2}=\sum_{n=2}^{\infty }\frac{e^{-a_{n}\tau }\left(1+a_{n}\tau \right)}{{\left(2n+1\right)}^{2}}\;\;\mathrm{and}\;\;R_{1}=\sum_{n=2}^{\infty }\frac{a_{n}e^{-a_{n}\tau }}{{\left(2n+1\right)}^{2}}=D\frac{\pi^{2}}{L^{2}}\sum_{n=2}^{\infty }e^{-a_{n}\tau },$$

we have *R*(*t*) ≈ *R*_2_ − *R*_1_*t*, then the equation for the moisture content is

(16) $$\mathrm{MR}\approx \frac{8}{\pi^{2}}\left\{\exp\left\{-a_{0}t\right\}+\frac{1}{9}\exp\left\{-9a_{0}t\right\}+R_{2}-R_{1}t\right\}$$

This equation gives a guideline to approximately find the diffusion coefficient. We propose to use the decay time *τ* = *a*_2_ and *n* = 2. By nonlinear regression, we found *a*_0_ and *R*_1_ that were compared with theoretical results to determine the diffusion coefficient. The condition *M*(0) = *M*_0_ makes *R*_2_ = *R*(0) = *π*^2^⁄8 − 10/9 ≈ 0.1226. In fact, Equation (16) is an extended case of the model proposed by Midilli et al. [24]. We highlight that the coefficients of our proposal have physical meaning.

#### 3.4.2. Temperature-Dependent Heuristic Model

To build a temperature-dependent moisture ratio model, we assume an effective diffusion coefficient that linearly depends on temperature. By developing the Taylor series expansion of Equation (3) we have

(17) $$D\left(t\right)\approx D_{A}\exp\left\{-\frac{\theta_{A}}{\theta_{0}}\right\}\left(1+\frac{\theta_{A}}{\theta_{0}^{2}}\left(\theta -\theta_{0}\right)\right)$$

where *q* is the sample temperature in Kelvin. This assumption was based on the experimental results of effective diffusion published by Sahin et al., where authors founded a quasilinear behavior of effective diffusion in the temperature range 40–60 °C [16]. We substitute in the Equation (16) and, after some mathematical arrangements, we obtain a new model that explicitly depends on temperature:

(18) $$\mathrm{MR}\approx \frac{8}{\pi^{2}}\left\{\exp\left\{-R_{3}f\left(\theta \right)t\right\}+\frac{1}{9}\exp\left\{-9R_{3}f\left(\theta \right)t\right\}+R_{2}-R_{1}t\right\}$$

where

(19) $$R_{3}=\pi^{2}L^{-2}D_{A}\exp\left\{-\frac{\theta_{A}}{\theta_{0}}\right\}$$

and

(20) $$f\left(\theta \right)=1+\frac{\theta_{A}}{\theta_{0}}\left(\frac{\theta }{\theta_{0}}-1\right).$$

**Figure 10:**
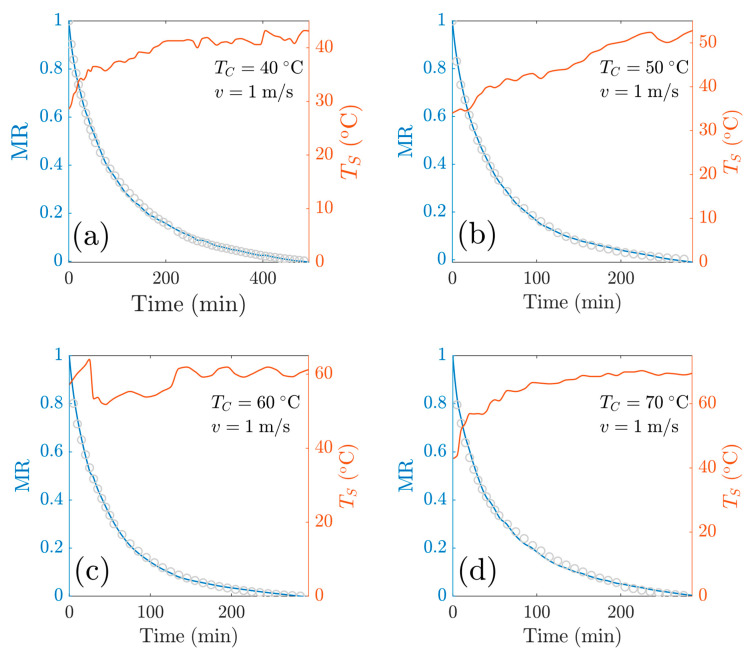
Reduced moisture content as a function of time at *v* = 1 m/s. Comparison of experimental data with the Heuristic+$R_{1}$+θ model: at a central temperature of (**a**) *T_c_* = 40 °C; (**b**) *T*_c_ = 50 °C; (**c**) *T_c_* = 60 °C; (**d**) *T_c_* = 70 °C.

**Figure 11:**
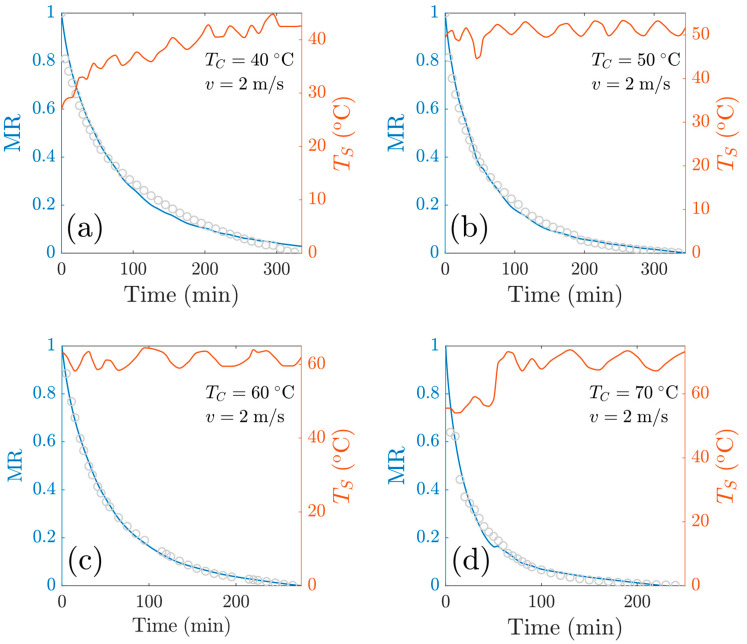
Reduced moisture content as a function of time at *v* = 2 m/s. Comparison of experimental data with the Heuristic+$R_{1}$+θ model: at a central temperature of (**a**) *T_c_* = 40 °C; (**b**) *T_c_* = 50 °C; (**c**) *T*_c_ = 60 °C; (**d**) *T_c_* = 70 °C.

This temperature-dependent model consists of three fitting parameters *R*_1_, *R*_3_ and *q_A_*/*q*_0_. Figure 10 shows both the fitting curves for the experiments at *v* = 1 m/s as well as the sample temperature. Figure 11 shows both the fitting curves for experiments at *v* = 2 m/s as well as the sample temperature. Overall, the model has comparable goodness of fit with models M1 and M2, as shown in Table A2, Table A3 and Table A4. The goodness-of-fit statistics for the different mathematical models used in this study are presented in Appendix A.

For the experiments at *v* = 1 m/s, the RMSE values were as follows: 0.0074 (*T_c_* = 40 °C), 0.0076 (*T_c_* = 50 °C), 0.0101 (*T_c_* = 60 °C) and 0.0133 (*T_c_* = 70 °C), and for experiments at v=2 m/s, the RSME values were 0.0216 (*T_c_* = 40 °C), 0.0207 (*T_c_* = 50 °C), 0.0091 (*T_c_* = 60 °C) and 0.0278 (*T_c_* = 70 °C). From these data, we observe the models show a great capacity to predict the drying kinetics.

## 4. Conclusions

In this work, mathematical models were implemented to predict the convective drying of goat meat. The experimental data set was compared with thin layer models and a heuristic approach. Our main findings are the following:

- According to our experimental results, the effect of air temperature on drying kinetics is very important. We observe the drying time decreases as temperature increases. For experiments at *v* = 1 m/s, we can observe that after 290 min, the samples subjected to *T_c_* = 40 °C reached a moisture content of 0.26 (d.b), at *T_c_* = 50 °C, a value of 0.22 (d.b), at *T_c_* = 60 °C, a value of 0.18 (d.b) and for the conditions at *T_c_* = 70 °C, a value of 0.07 (d.b). The temperature causes a very significant reduction in drying time. This was also corroborated by analyzing the drying rate curves, since it was observed that the initial drying rate for the drying condition at *T_c_* = 70 °C was practically twice the rate at *T_c_* = 40 °C. Furthermore, for experiments at 1 m/s, the constat drying rate was not observed.
- Regarding the drying kinetics for the four temperatures at *v* = 2 m/s, a second drying rule was confirmed: As airflow velocity increased, the drying rate increased. The final moisture content for this set of experiments was lower with respect to the experiments performed at *v* = 1 m/s; in fact, all the samples reached moisture contents below 0.2 (d.b). For these drying conditions, the falling drying rate period was always identified.
- Regarding the mathematical models, it was observed that they described the drying kinetics’ behavior with an accurate fitting of experimental data. The two-term models, the Midilli model and the Wang and Singh model showed very small RMSE values for all drying conditions. Also, the magnitude of the diffusion coefficients corresponded to the usual values for biological materials (~10^−9^ m^2^ s^−1^), which indicates the reliability of our calculations. In relation to the heuristic model, the drying curves for the four temperatures also showed a good prediction of data. This model considered the effect of temperature oscillation, which allows us to consider the thermal dynamics of the process.

## Figures and Tables

**Table 1:** Mathematical drying models used in the current study.

Model	Name	Equation		References
M1	Two-term	$\mathrm{MR}=a\exp\left(-k_{1}t\right)+b\exp\left(-k_{2}t\right)$		[20]
		Special case	Two-term exponential: $b=1-a,\;k=k_{1},\;k_{2}=k_{1}a$	[21]
			Diffusion: $b=1-a,\;k=k_{1},\;k_{2}=k_{1}b$	[22]
			Verma et al.: $b=1-a,\;k=k_{1},\;k_{2}=g$	[23]
M2	Midilli (Extended)	$\mathrm{MR}=a\exp\left(-kt^{n}\right)+bt^{m}$		[24]
			Logarithmic: n=1, m=0	[25]
			Henderson and Pabis: n=1, b=0	[26]
		Special case	Page: a=1, b=0	[27]
			Modified Page: $a=1,\;b=0,\;k\to k^{n}$	[27]
			Newton/Lewis: a=1, b=0, n=1	[27,28]
M3	Polynomial	$\mathrm{MR}=a_{0}+a_{1}t^{1}+a_{2}t^{2}+\cdots +a_{N-1}t^{N-1}+a_{N}t^{N}$		
		Special case	Wang and Singh: $a_{0}=1,\;a_{1}=a,\;a_{2}=b,\;a_{k}=0\;\forall k>2$	[28]
M4	Heuristic+$R_{1}$	$\mathrm{MR}=\frac{8}{\pi^{2}}\left\{\exp\left\{-a_{0}t\right\}+\frac{1}{9}\exp\left\{-9a_{0}t\right\}+R_{2}-R_{1}t\right\}$		Current study
		with $R_{2}=0.1226$		
M5	Heuristic+$R_{1}$+θ	$\mathrm{MR}=\frac{8}{\pi^{2}}\left\{\exp\left\{-R_{3}f\left(\theta \right)t\right\}+\frac{1}{9}\exp\left\{-9R_{3}f\left(\theta \right)t\right\}+R_{2}-R_{1}t\right\}$		Current study
		with $R_{2}=0.1226\;$, $f\left(\theta \right)=1+\frac{\theta_{A}}{\theta_{0}}\left(\frac{\theta }{\theta_{0}}-1\right)$.		

**Table 2:** Mathematical meat drying models used in the current study restricted to MR(*t*=0) = 1. Heuristic models satisfy this condition.

Model	Name	Equation	Parameter
M1	Two-Term	$\mathrm{MR}=\left(1-b\right)\exp\left(-k_{1}t\right)+b\exp\left(-k_{2}t\right)$	a=1−b
M2	Midilli (Extended)	$\mathrm{MR}=\exp\left(-kt^{n}\right)+bt^{m}$	a=1
M3	Polynomial	$\mathrm{MR}=1+a_{1}t^{1}+a_{2}t^{2}+\cdots +a_{N-1}t^{N-1}+a_{N}t^{N}$	$a_{0}=1$

**Table 3:** Diffusion coefficient *D* (10^−9^ m^2^ s^−1^).

Air Velocity	Coefficients	Central Drying Temperature *T_c_* (°C)			
		40	50	60	70
$1\;\frac{m}{s}$	$k_{1}/k_{2}$	0.15	0.11	0.08	0.11
	b	0.77	0.80	0.82	0.71
	D	7–20	14–40	16–44	12–33
$2\;\frac{m}{s}$	$k_{1}/k_{2}$	0.06	0.11	3.94	0.11
	b	0.77	0.74	0.28	0.62
	D	0.9–3	1–3	5–15	2–6

## Data Availability

The original contributions presented in the study are included in the article/Appendix A, further inquiries can be directed to the corresponding author.

## Appendix A

For the heuristic, two-term and Midilli models, the Curve Fitting Toolbox of MATLAB was used for parameter optimization by means of nonlinear regression. For the Wang and Singh model, the POLYFIT instruction of MATLAB was used. This instruction calculates the polynomial coefficients by means of linear regression. Tables with statistical information are shown below. The standard error (RMSE, root mean square error) of the fits to the different models ranges in the interval (0.004, 0.029). Table A1 presents the values concerning the validation of the regression. Furthermore, Table A2, Table A3, Table A4 and Table A5 have information related to the values of the fit parameters as well as the goodness of fit.

**Table A1:** Definitions for regression validation.

Field	Value	Description
RSS	$\overset{n}{\underset{i=1}{\sum }}{\left(y_{i}-{\hat{y}}_{i}\right)}^{2}$	Sum of squares due to error or residual sum of squares. Here ${\hat{y}}_{i}$ is the estimator and n is the sample size.
ESS	$\overset{n}{\underset{i=1}{\sum }}{\left({\hat{y}}_{i}-\bar{y}\right)}^{2}$	Explained sum of squares. Here, $\bar{y}$ is the arithmetic mean.
TSS	$\overset{n}{\underset{i=1}{\sum }}{\left(y_{i}-\bar{y}\right)}^{2}$	Total sum of squares.
RSQUARE ($R^{2}$)	$\frac{\mathrm{ESS}}{\mathrm{TSS}}=\frac{\mathrm{TSS}-\mathrm{RSS}}{\mathrm{TSS}}$	R-squared (coefficient of determination).
DFE	n−k−1	Degrees of freedom in the error, where k is the number of predictors.
ADJRSQUARE	$1-\left(1-R^{2}\right)\frac{n-1}{n-k-1}$	Degree-of-freedom adjusted coefficient of determination.
RMSE	$\sqrt{\frac{1}{n}\overset{n}{\underset{i=1}{\sum }}{\left(y_{i}-{\hat{y}}_{i}\right)}^{2}}$	Root mean square error (standard error).

**Table A2:** Temperature-dependent heuristic model.

Air Velocity		Coefficients *			Goodness				
	$T_{c}$	$R_{3}$	$R_{1}$	$\theta_{A}/\theta_{0}$	RSS	RSQUARE	DFE	ADJRSQUARE	RMSE
1 m/s	40	0.0093	0.0003	−1.4130	0.0029	0.9993	52	0.9993	0.0074
	50	0.0164	0.0005	−1.1000	0.0018	0.9993	32	0.9992	0.0076
	60	0.0256	0.0004	0.4998	0.0033	0.9986	33	0.9986	0.0101
	70	0.0156	0.0004	−2.0000	0.0058	0.9974	33	0.9974	0.0133
2 m/s	40	0.0083	0.0006	−3.3720	0.0172	0.9935	37	0.9993	0.0216
	50	0.0188	0.0004	−0.7729	0.0164	0.9939	38	0.9936	0.0207
	60	0.0206	0.0004	−0.2490	0.0026	0.9990	31	0.9989	0.0091
	70	0.0347	0.0006	−1.3720	0.0246	0.9854	32	0.9845	0.0278

* 95% confidence bounds.

**Table A3:** Two-term model (M1).

Air Velocity		Coefficients *			Goodness				
	$T_{c}$	b	$k_{1}$	$k_{2}$	RSS	RSQUARE	DFE	ADJRSQUARE	RMSE
1 m/s	40	0.2240	0.0082	0.0565	0.0019	0.9995	52	0.9995	0.0061
	50	0.1910	0.0158	0.1459	0.0009	0.9996	32	0.9996	0.0053
	60	0.1830	0.0172	0.2165	0.0015	0.9994	33	0.9994	0.0068
	70	0.2923	0.0131	0.1230	0.0037	0.9984	32	0.9983	0.0107
2 m/s	40	0.2297	0.0097	0.1545	0.0077	0.9971	37	0.9969	0.0144
	50	0.2639	0.0128	0.0128	0.0022	0.9992	38	0.9991	0.0076
	60	0.7201	0.0578	0.0147	0.0013	0.9995	31	0.9995	0.0065
	70	0.3808	0.0226	0.2104	0.0105	0.9938	32	0.9934	0.0181

* 95% confidence bounds.

**Table A4:** Midilli model (M2).

Air Velocity		Coefficients *			Goodness				
	$T_{c}$	b	k	n	RSS	RSQUARE	DFE	ADJRSQUARE	RMSE
1 m/s	40	−0.0001	0.0318	0.7626	0.0012	0.9997	52	0.9997	0.0047
	50	−0.0001	0.0505	0.7658	0.0005	0.9998	32	0.9998	0.0041
	60	−0.0001	0.0548	0.7629	0.0015	0.9994	33	0.9993	0.0068
	70	−0.0002	0.0781	0.6482	0.0007	0.9997	32	0.9997	0.0048
2 m/s	40	−0.0003	0.0667	0.6148	0.0012	0.9996	37	0.9995	0.0057
	50	−0.0001	0.0632	0.6933	0.0011	0.9996	38	0.9996	0.0054
	60	−0.0000	0.0594	0.8267	0.0022	0.9991	31	0.9991	0.0084
	70	−0.0001	0.1372	0.6325	0.0096	0.9943	32	0.9940	0.0173

* 95% confidence bounds.

**Table A5:** Wang and Singh model (M3).

Air Velocity				Coefficients *			Goodness				
	$T_{c}$	$a_{4}$	$a_{3}$	$a_{2}$	$a_{1}$	$a_{0}$	RSS	RSQUARE	DFE	ADJRSQUARE	RMSE
1 m/s	40	0.0463	−0.0980	0.0618	−0.1124	0.1267	0.0728	0.9953	50	0.9949	0.0186
	50	0.0488	−0.1173	0.0861	−0.1114	0.1167	0.0712	0.9947	30	0.9940	0.0195
	60	0.0535	−0.1285	0.0829	−0.0840	0.0946	0.0681	0.9939	30	0.9928	0.0205
	70	0.0601	−0.1211	0.0409	−0.0963	0.1487	0.0643	0.9903	30	0.9890	0.0251
2 m/s	40	0.0459	−0.0946	0.0415	0.1300	0.1955	0.0658	0.9931	35	0.9923	0.0215
	50	0.0567	−0.1166	0.0530	0.0881	0.1108	0.0647	0.9898	36	0.9886	0.0259
	60	0.0530	−0.1264	0.0843	0.1058	0.1242	0.0753	0.9967	36	0.9970	0.0157
	70	0.0633	−0.1509	0.0571	0.0571	0.0639	0.0463	0.9588	30	0.9533	0.0446

* 95% confidence bounds.
